# A novel approach for simulating and optimizing the production costing system

**DOI:** 10.1016/j.heliyon.2024.e40932

**Published:** 2024-12-04

**Authors:** Mohamed O. Hegazi

**Affiliations:** Department of Computer Science, College of Computer Engineering and Science, Prince Sattam Bin Abdulaziz University, Al-Kharj, Saudi Arabia

**Keywords:** Simulations, Optimization, Linear programming, Production cost, Computer system

## Abstract

This paper presents a novel approach for simulating and optimizing production costing systems using linear programming. The proposed method employs a linear programming algorithm to simulate the behavior of production costs and to derive optimal solutions, including cost minimization, resource maximization, and economic returns. This approach has been implemented as a standard platform computer application, which automatically processes the costing system to reflect real-world behavior accurately. The developed application was tested on a university college costing system. The results demonstrated that the approach effectively simulates production costing systems and provides comprehensive reports and results, such as optimal costs, efficient resource utilization, and profit calculations. All processes are stored automatically in a database, supported by a full user interface with forms for ease of use. This approach is valuable for controlling costs and aiding decision-making processes. The versatility and efficiency of the linear programming model, particularly in handling large datasets and linear relationships, make it a powerful tool in various sectors.

## 1 Introduction

Achieving the optimal allocation of costs and production inputs is crucial for the success of most productive institutions, whether they provide industrial, agricultural, or human services products. The cost of producing a product or service is directly related to the quantity of inputs required. As the number of products increases, the quantity of inputs also rises [1]. This relationship prompts the question: how can we determine the optimal cost limit and achieve the best use of production inputs?

Simulating and optimizing the production cost system play a pivotal role in businesses by enabling comprehensive cost analysis and informed decision-making. Through the simulation of the production process, companies can gain valuable insights into their cost structures, identify inefficiencies, and pinpoint areas needing improvement. This facilitates more accurate estimation of production costs, allowing businesses to allocate resources efficiently and minimize unnecessary expenses. Additionally, optimizing the production cost system ensures that businesses operate at peak efficiency, maximizing output while minimizing costs. This not only enhances profitability but also improves competitiveness in the market. Furthermore, by continuously monitoring and optimizing the production cost system, businesses can adapt to changing market conditions and emerging trends, remaining agile and responsive in dynamic environments. Ultimately, the ability to simulate and optimize the production cost system empowers businesses to make strategic decisions that drive growth, reduce costs, and enhance overall performance.

Linear programming (LP) is a mathematical optimization technique used to achieve the best outcome in a given mathematical model. Its applications in production cost optimization are well-documented. Linear programming helps in determining the most cost-effective allocation of limited resources while meeting required constraints. By setting up a linear objective function and subjecting it to various linear constraints, organizations can identify optimal production levels, resource allocation, and cost minimization strategies [2,3]. LP has been successfully applied in numerous fields, including manufacturing, transportation, and finance, making it a versatile tool for cost optimization [4].

Simulation involves creating a digital twin of the production process to model its behavior under different conditions. It allows for the analysis of complex systems that are difficult to represent mathematically with traditional optimization techniques. By simulating various scenarios, businesses can observe potential outcomes, identify bottlenecks, and evaluate the impact of different strategies on production costs. Simulation models can be particularly useful in understanding stochastic and dynamic systems where variability and uncertainty are significant factors [5].

There are diverse approaches to combining simulation and optimization techniques for problem-solving, including Simulation Purpose, Hierarchical Structure, Search Method, and Search Scheme. Simulation and optimization methodologies, along with their integration approaches, demonstrate the synergistic benefits of leveraging detailed insights from simulation models alongside the computational power of optimization techniques. This taxonomy serves as a valuable guide for researchers navigating the landscape of simulation-optimization methods, fostering a standard for communication within the scientific community and paving the way for future advancements in the field of operational research [6]. Combining linear programming with simulation provides a comprehensive approach to production cost optimization. Linear programming offers precise optimization within defined constraints, while simulation provides insights into the variability and dynamic behavior of production processes. This integrated approach enables more accurate and reliable decision-making, ensuring that the optimized solutions are not only theoretically sound but also practically feasible in real-world scenarios [7,8].

Research has shown that this combined approach can lead to substantial cost savings and efficiency improvements. For instance, the integration of these methods has been applied in industries ranging from automotive manufacturing to service operations, demonstrating their effectiveness in reducing costs and improving productivity [9].

This paper presents an automated model based on linear programming that functions as an automatic template to find optimal costs and the best use of production inputs through simulating the production system behavior. The paper also presents an experiment with the model in calculating the costs of higher education institutions.

The rest of this paper is structured as follows: Section 2 presents the related work, investigating research in the area of cost behavior simulation and optimization. In Section 3, we present the proposed model. Section 4 details the experiment. Section 5 discusses the results. Section 6 presents the evaluation study. Finally, conclusions are reported.

## 2 Related work

Recently, many research works have focused on studying simulation modeling and optimization cost in diverse industrial and environmental contexts (Table 1). In Rosova et al. [10], the authors present a case study on utilizing computer simulation modeling to enhance the efficiency and performance of custom production processes, emphasizing the importance of simulation in identifying bottlenecks and improving business performance. Similarly, Schneider et al. [11] develop an online simulation model to estimate the total costs of tobacco product waste in large U.S. cities, providing valuable insights for policymakers to address the economic and environmental impacts of tobacco product litter. Khzouz et al. [12] investigate life cycle costing analysis as a tool for estimating hydrogen production costs for fuel cell vehicle technology, highlighting the economic feasibility of different production methods. Atalan [13] conducts a cost analysis using discrete-event simulation in nurse and doctor employment management, demonstrating the effectiveness of simulation in evaluating treatment costs and resource allocation. Kristiana et al. [14] design a simulation model to improve the production efficiency of the batik industry, showcasing the potential of simulation for enhancing manufacturing processes in small-medium enterprises. Trilaksono and Laksono (2022) apply simulation modeling to address production inefficiencies in the garment industry, demonstrating how simulation can optimize production flow and increase productivity. Zhang et al. [15] compare the techno-economic viability of green ammonia production processes, underscoring the importance of sustainable manufacturing practices. Parv et al. [16] propose a simulation model for sustainable manufacturing systems, integrating economic and environmental factors to minimize costs and energy consumption. Jordan et al. [17] introduce simulation of cost-driven value stream mapping, providing insights into cost-efficient production strategies and process optimization. Bhatt and Tao [18] provide a comprehensive analysis of the economic perspectives of biogas production via anaerobic digestion, highlighting the potential for this technology to contribute to sustainable energy solutions. Their study in Bioengineering examines the cost structures, market dynamics, and financial incentives associated with biogas production, underscoring the importance of integrating economic and environmental factors in the development of renewable energy projects their work aligns with the broader objective of minimizing costs and energy consumption in sustainable manufacturing systems. Stevanov et al. [19] optimize the subassembly production process using simulation, focusing on minimizing cycle time and maximizing output quantity. Shafipour-Omrani et al. [20] develop a simulation-optimization model for liquefied natural gas transportation, considering product variety to reduce transportation costs. Rivera-Gómez et al. [21] propose a joint optimization model for production and maintenance strategies, incorporating dynamic sampling strategies to minimize costs and meet quality constraints in deteriorating systems. Ourya et al. [22] present an evaluation for the technical and economic feasibility of harnessing renewable resources to produce hydrogen, offering a comprehensive analysis of the potential and challenges associated with such systems. Their work particularly in optimization the renewable energy systems and development of sustainable manufacturing practices. Adeleke et al. [23] explore the future of precision manufacturing, emphasizing the role of these technologies in achieving higher accuracy and efficiency, their study highlights the benefits of combining sophisticated measurement techniques with real-time monitoring to optimize manufacturing processes and ensure product quality. Finally, Liu et al. [24] investigate an agent-based simulation and optimization approach for hybrid flow shops, specifically considering the impact of multi-skilled workers and fatigue factors, their study provides a detailed analysis of how worker versatility and fatigue can affect production schedules and outcomes.

**Table 1:** A summary of the related work.

Study	Focusing	Methodology
Rosova et al. [10]	Improving efficiency and performance of production processes. Custom production processes, efficiency, and performance using simulation	Conducted a case study using simulation modeling to identify bottlenecks and improve business performance. They used EXTENDSIM 9 software
Schneider et al. [11]	Estimating total costs of tobacco product waste in large US cities	Developed an online simulation model using Monte Carlo simulation techniques to estimate costs, providing insights for policymakers.
Khzouz et al. [12]	Determining hydrogen production cost for fuel cell vehicle technology	Utilized life cycle costing analysis to estimate production costs for hydrogen production.
Atalan [13]	Analyzing costs in nurse and doctor employment management	Employed discrete-event simulation to evaluate treatment costs and resource allocation.
Kristiana et al. [14]	Enhancing production efficiency of the Batik industry	Designed a simulation model to improve manufacturing processes in small-medium enterprises.
Trilaksono and Laksono [28]	Preparing supply in the garment industry through capacity using simulation model	Utilized simulation modeling to address production inefficiencies and optimize production flow, using Promodel 16 simulation software
Zhang et al. [15]	Comparing green ammonia production processes	Conducted a techno-economic comparison to assess the viability of different production processes, using
Parv et al. [16]	Modeling sustainable manufacturing systems	Developed a simulation model integrating economic and environmental factors for sustainable systems. Detailed modeling and optimization
Jordan et al. [17]	Analyzing cost-driven value stream mapping in production	Introduced simulation of cost-driven value stream mapping to identify cost-efficient production strategies.
Bhatt and Tao [18]	Investigating economic perspectives of biogas production via anaerobic digestion	Conducted an economic analysis examining the potential contributions of biogas production to sustainability. They used Aspen Plus process modeling and CapdetWorks software
Stevanov et al. [19]	Optimizing the subassembly production process	Employed simulation to optimize the subassembly production process, focusing on minimizing cycle time.
Shafipour-Omrani et al. [20]	Developing a simulation-optimization model for liquefied natural gas transportation	Developed a simulation-optimization model considering product variety to reduce transportation costs.
Rivera-Gómez et al. [21]	Joint optimization of production and maintenance strategies for deteriorating systems	Proposed a joint optimization model incorporating dynamic sampling strategies to minimize costs.
Ourya et al. [22]	Assessing green hydrogen production using hybrid renewable sources	Evaluated the technical and economic feasibility of green hydrogen production, particularly in Morocco.
Adeleke et al. [23]	Exploring the future of precision manufacturing	Explored the integration of advanced metrology and intelligent monitoring for process optimization.
Liu et al. [24]	Agent-based simulation and optimization of hybrid flow shop considering worker factors	Utilized agent-based simulation and optimization to analyze the impact of worker factors on production outcomes, focusing on fatigue.

## 3 The proposed approach

**Fig. 1:**
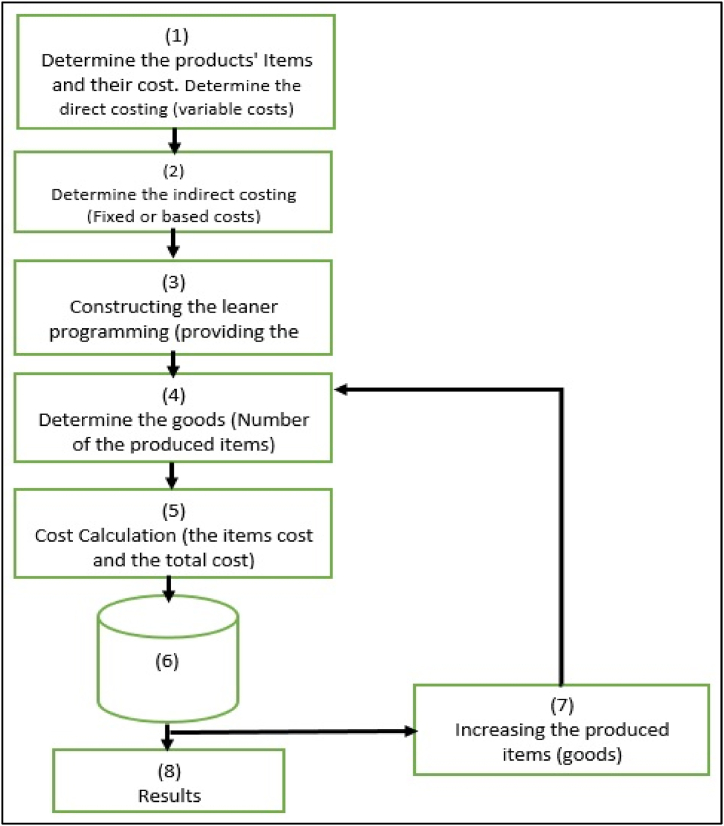
The framework of the suggested approach.

The suggested approach integrates the optimization methods with the simulation methods in one computer system. Therefor this approach provides the methodology of developing a computer application that implements the integration of these two methods in one automated platform in which cost behavior is simulated and provided cost optimization results (Fig. 1).

As it is shown in figure (1) the approach contains of eight modules working as follow.

1 **In the first module**, the direct costing (variable costs) of the product or service are determined by identifying the direct cost elements in the production the product or the service, and then determining the cost of each element (for example P, C_i_ and X_i_, where, P refers to the product, C_i_ for element i in the product P, and X_i_ refers to the cost of that element).
2 **In the second module**, the fixed or the based costing (indirect costs) are determined by calculating the depreciation value of buildings, utilities, offices, equipment, furniture, etc., along with the management and service costs.
3 **In the third module**, the relationship between the produced unit and the required quantity of each cost element is determined and put in the form of a linear equation as follows:

(1) $$C_{i}=Round\left(P\times R_{i}\right)$$

(2) $$TotalCost=\sum_{i=1}^{n}C_{i\;}\times X_{i}$$

(3) $$UnitCost=\frac{TotalCost}{P}$$

**P**: represented the product (the produced item –good).

**R**_**i**_: represented the element ratio of the product (Ri), (because each product is produced by combining many items (Ci) and each item has ratio that indicate his participation in the producing the product).

**C**_**i**_: represent the element (numbers of items).

**X**_**i**_: represented the element cost (item cost).

In equations (1), (2), (3), as ***C***_***i***_ represents the constituent elements of production, it starts with an initial value then it increases with the increasing of the number of variables. This aspect represents the automatic variable of the elements, as it is the mechanism on which the model relies in calculating the best use of the elements of production. As shown in equation (1), the value of ***C***_***i***_ is calculated by multiplying the product ***P*** by the element ratio of the product ***R***_***i***_. Round function has been used to get rid of fractions, because elements do not accept fractions.

4 **In the fourth module**, the number of products, that will be produced, were determined. The determination begins with an initial value and then automatically increased, following the cost behavior (equation (1)), till reaching the optimal values of the cost or the profit, also can be monitoring by the application's user if he wants to examine certain scenario
5 **In the fifth module**, the costs values are determined by calculating the total cost of the products (equation (2)) and unit cost (equation (3)). Also many statistical results in this regards will be produced, such as average, min-max values … etc.
6 **In the sixth module**, the results, that provided by the fifth module, will be saved in the database (i.e. this module is about all the process that concerning with database including designing, implementation, and processing).
7 **In the seventh module**, the products are increased automatically as the steps are repeated from step four, and with the increase in the number of products, the production elements ***C***_***i***_ increase according to the coefficients ***R***_***i***_, as it shown in the linear equations (1), (2). In this module the exception on constrains (constrains of the constrain) well be handling. The approach takes into account a permissible rate of the products increase without resorting to an increase in the cost (exception on constrains). For example, in university, if the rule says that when the number of students exceeds 40 students, we need a new lecture room, however it is possible to allow the increase of students to reach, for example, 45 students without adding a new lecture room. This aspect represents the way in which our approach provides the maximization of resource used.
8 **In the last module (the eighth module)**, the finals results are provided. Bases one database concepts, it will be possible to extract results that show the cost, the optimal number of the production inputs, and provided varieties results for help in making dissection such as examine some scenarios.

## 4 Model designing and implementation

This section presents the practical implementation of our approach. The approach will be applied in developing a model for higher education costing system. The model will simulate the higher education costing system and provide optimum use of the resource and the optimum cost.

### 4.1 Model designing

Higher education institutions are considered as a suitable field for applying this approach because the elements of the academic process in higher education institutions are characterized by their overlapping with each other, which causes many issues that require ideal solutions such as.

1. How can these institutions determine the appropriate number of lecture rooms, laboratories and other building (maximizing use of resource)? without being charged with unneeded supplies (optimal use of infrastructure - fixed costs)
2. How can these institutions determine the appropriate number of faculty members, assistant staff, administrators, workers, etc.? In order to not use redundant workers, which results an increase in the cost. (Optimal use of human resources and minimizing the cost-production inputs)
3. What is the constrains and their effective relationships in such institutions? As example:
a) What is the relationship of these elements to the number of students?
b) How the maximum capacity of institutions can be determined? Is it according to the number of students, lecture rooms, laboratories, professors … etc? And what does these have to do with the various cost elements?
c) What is the relationship between the academic period and the fiscal year? Does this have an impact on costing operations?
d) The constrains on using assistants staff's costs (technicians, tutors, and teaching assistants).
e) The effect of the other costs such as auxiliary equipment and teaching aids in minimizing the cost and optimizing the resources.

Accordingly, for developing the computer application three requirements must be stated, these requirements are:

**First:** Specifying the direct items and their costs (the direct costs (variable costing)) such as.

1 Teaching staff and their costs.
2 Classrooms and their costs (annual consumption + expenses).
3 Laboratories and their costs (annual consumption + expenses).
4 Assistant's staffs and their costs (lab attendants, technicians and teaching assistants).
5 Costs of auxiliary equipment and teaching aids.

**Second:** Specifying the indirect items and their costs (the fixed costs (indirect costing)) which is represents in the costs of administration, services, libraries, and buildings. Bearing in mind that these costs are differ according to the nature or the study's type (theoretical - scientific - practical (training), etc.). Those items even they have fixed cost but according to the nature of higher education they are not static because they changed dynamically according to certain level. For example, the costs of administration, services, and the value of consumption of buildings increase when the number of students exceeds a specified limit, or when academic periods are extended. In general, the change in the costs of higher education institutions is associated with the change of two factors.

1. The number of students (the number of students can be likened to the product of an educational institution)
2. The studying period during the day or the week (the period can be likened to production capacity - or production lines)

The relationship between the changes in the cost of items takes a special approach, for example when the increase of the number of students reaches a certain limit, then the item costs increase in a different level may be depends on other items (and this is the reality of all costs of industrial, agricultural or service products). For example, increasing the studying period may achieve an optimal use of lecture rooms and laboratories, but it may lead to an increase in the number of professors and the rest of the elements that similar to this item, and so on.

**Third**: Specifying the constrains, in higher education there are specific rates control the change in the elements of costs and the factors that affects them. These rates are.

1. A student-to-faculty ratio. (Faculty member rate for students)
2. Student/lab rate
3. Lecture rooms rate for students (student/lecture_ room)
4. A student-to-assistant staff ratio. (The assisting staff rate (technicians - teaching assistants - assistants) for students).
5. The rate of use of lecture rooms and laboratories during the study period.

These requirements can be putted in variables as follow.

• ***t***: for the total cost, and it represents the sum of the variable costs and fixed costs.
• ***S***: for the number of students (it will be initialized first then automatically incremented)
• ***x***_***1***_: for the cost of a faculty member, and ***c***_***1***_: for the numbers of faculty members.
• ***x***_***2***_: for of the laboratory, and ***c***_***2***_: the numbers of laboratory.
• ***x***_***3***_: for lecture rooms cost and ***c***_***3***_: numbers of Lecture rooms.
• ***x***_***4***_: for the cost of the assistant, technician, or tutors (average), and ***c***_***4***_: the numbers of the assistant staff.
• ***x***_***5***_: for the fixed costs (administrative costs and auxiliary costs) and ***c***_***5***_: fixed cost coefficient (we used coefficient because these costs do not depend on the number, but are calculated during the financial period, so the value of this coefficient = 1 in the event that the available capacity for work is not exceeded. If the maximum capacity is exceeded, then additional expansions will be needed at specific rates, and in this case the fixed costs increase according to the expansions, because of that the value of ***c***_***5***_ changes at the rate of this increase (1 + percentage of increase)).

The constrains values R_1_, R_2_, R_3_ and R_4_ represent the usage rates of each of the elements and S_1_, S_2_, S_3_, and S_4_ (the corresponding number of students for each constrain), as follow.

• ***R***_***1***_: Ratio of teaching staff to students: R_1_= (1/S_1_), where ***S***_***1***_ is the number of students the professor has.
• ***R***_***2***_: the rate of assistants for students: R_2_= (1/S_2_), where ***S***_***2***_ is the number of students the assistant has.
• ***R***_***3***_: the rate if lecture rooms rate for students: R_3_= (1/S_3_), where ***S***_***3***_ is the capacity of the lecture room (Students number)
• ***R***_***4***_: the rate of the laboratory for students: R_4_= (1/S_4_), where ***S***_***4***_ is the capacity of the laboratory.

Other constrains values, such as the operating periods of lecture rooms and laboratories were taken into account, because when working period of the lecture rooms and laboratories increases the costs will decrease (as the lecture room accommodates other student groups), in this manner the optimal use of both the lecture rooms and the laboratories will be achieved, and accordingly.

• ***M***_***n***_: the working periods for the lecture room.
• ***H***_***n***_: the working periods for the laboratory

As the work periods during the week is presented as ***n*** (one period can be measured in 2 h).

An addition constrains are needed to provide real simulation to behaviors of universities systems, where in the reality usually the number of students can increase out of limitation. The fallowing variables are used for handling these constrains (to provide the best maximization used of resources).

• ***U***_***1***_: The rate allowed to increase the number of students in the faculty member.
• ***U***_***2***_: The rate allowed to increase the number of students in the assistant staff.
• ***U***_***3***_: The rate allowed to increase the number of students within the laboratories.
• ***U***_***4***_: The rate allowed to increase the number of students in the lecture rooms.

### 4.2 Model implementation

**In the first step** in implementing the model, equations (1), (2), (3) were applied using the variable stated on the above section as follow:

$$c_{1}=Round\left(\left(S\times R_{1}\right)+\left(0.5-U_{1}\right)\right)\;c_{1}\geq 1$$

$$c_{2}=Round\left(\left(S\times R_{2}\right)+\left(0.5-U_{2}\right)\right)\;c_{2}\geq 1$$

$$c_{3}=Round\left(\left(S\times R_{3}\right)+\left(0.5-U_{3}\right)\right)\;c_{3}\geq 1$$

$$c_{4}=Round\left(\left(S\times R_{4}\right)+\left(0.5-U_{4}\right)\right)\;c_{4}\geq 1$$

$$c_{5}=1$$

$$Total\;cost\;t=c_{1}x_{1}+c_{2}x_{2}+c_{3}x_{3}+c_{4}x_{4}+c_{5}x_{5}$$

$$Student\;cost\;Sc=\frac{t}{S}\;S\to the\;numbers\;of\;students$$

$$The\;optimum\;cost=\min\left\{{Sc}_{i},{Sc}_{i+1},\ldots \ldots ..\;{Sc}_{record\;count}\right\}$$

Round function is used in performance all calculations to avoid obtaining fractions as the nature of the elements does not accept fractions, as it is impossible to say a professor and a half or a lecture room and a quarter, etc. Those numbers are determined automatically in our computer simulation. Since the Round function gives an opportunity to accept an increment of half of the total number, so in this model we will add to the value before calculating the function equivalent to the difference between the allowed rate and 0.5:(0.5 - U)

**Fig. 2:**
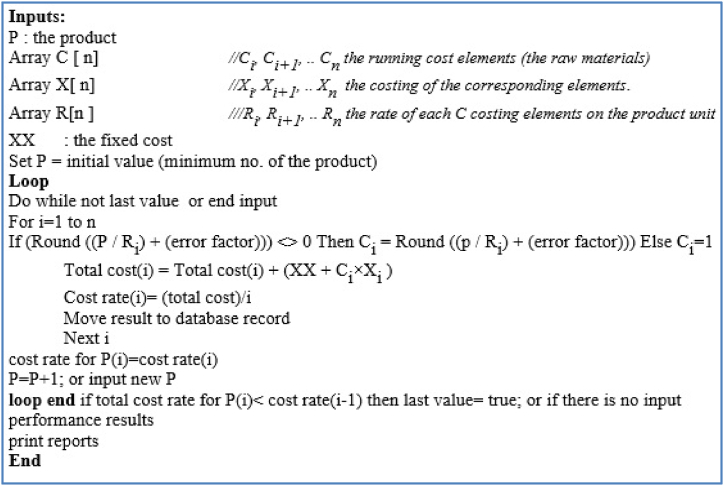
Model algorithm.

**In the second step**, the computer application will be developed based on the algorithm shown in Fig. 2. The algorithm is represented the pseudo code of the proposed approach Fig. 1.

**Fig. 3:**
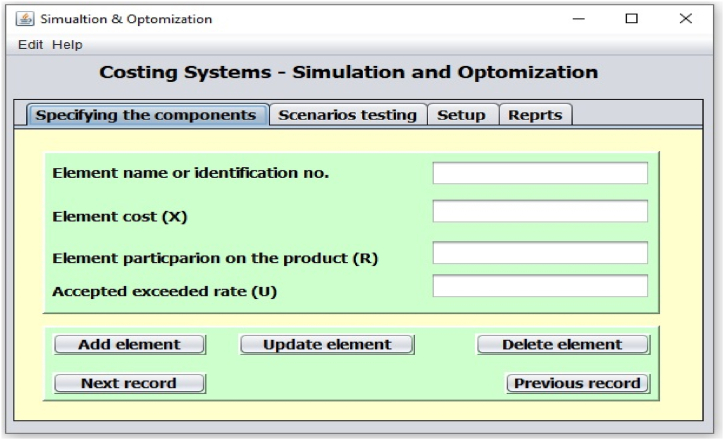
Sample of application's user interface.

The computer application presented the automation of the model with the benefit of using database system for storing and retrieves the model data. Pointing out that the automated model is a framework model that allows the user to specify elements and parameters through graphical user interfaces, as example Fig. 3.

## 5 Result and discussions

In this section, the model will be tested on an estimated numerical values that are closer to the reality of university colleges, and then present the results of the model.

Following are the estimated input values.

• Let us assume the flowing values for the constrains:
− R1 = 1/25 R2 = 1/20 R3 = 1/25 R4 = 1/20
− U1 = 0.25 U2 = 0.20 U3 = 0.25 U4 = 0.103)
− The study periods are 15 weeks (6 h per day for five days, 30 h per week) and the study period for the student is 12 weeks (24 h per week). Then:
− The rate of student's use of the lecture room = 12 × 75 % = 9
− The rate of using the lecture room by a batch of students = 9/15 = 0.6
− The rate of student's use of the teacher = 12 × 25 % = 3
− The rate of using the laboratory by a batch of students = 3/15 = 0.2
• and assume the flowing values for element costs:
− The average annual cost of a faculty member (X1) = 36,000 $
− The average annual cost of the assistant staff (X2) = 18,000 $
− The annual cost of a room with a capacity of 25 students (consumption or rent value) (X3) = 5000 $
− The annual cost of a 25-student laboratory (consumption or rent value) (X4) = 10,000 $
− The annual cost of administrative and service aspects (X5) = 20,000 $

**Fig. 4:**
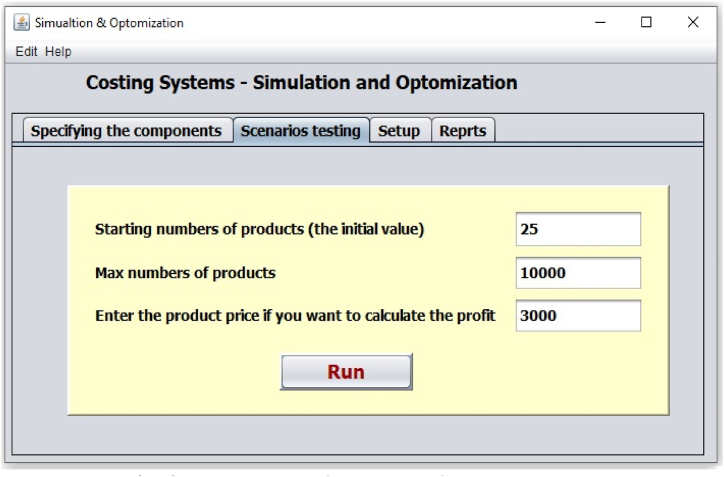
User interface for running the model (Scenario testing).

After estimated the values then these values will be entered into the application through the user interface input forms as it shown in Fig. 3. Because, the application was designed to provide an automatic generation, where it increases the number of students automatically and test the results at each number, and store the results into database, beside the ability of let the user makes test for any scenarios, so results can be obtained automatically after data was entering into the application, as example, when we run the application using the above assumed data and starting with 25 students to 10000 students (Fig. 4), the optimum student costs (2505.11 $) ($\min\left\{{Sc}_{i},{Sc}_{i+1},\ldots \ldots ..\;{Sc}_{record\;count}\right\}$) were achieved when the number of student reach 881 students, 35 faculty members, 44 assistants staff, 15 lecture rooms, and 6 laboratories, Table 2 showed a summary of some results comparing three values in respect to the number of students.

**Table 2:** Sample values from the results of the model.

Number of students	Faculty No.	Assistant staff No.	Lecture rooms No.	Labs. No.	Total costs	Student cost
25	1	2	1	1	107000	4280.00
881	35	44	15	6	2207000	2505.11
1000	40	50	17	7	2515000	2515.00

**Fig. 5:**
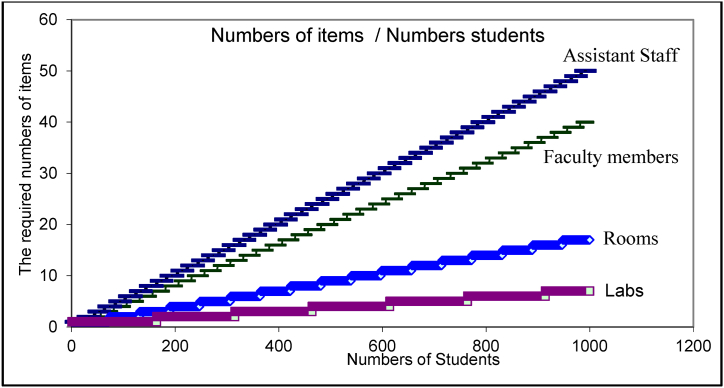
The simulating of the cost items behavior in university college.

**Fig. 6:**
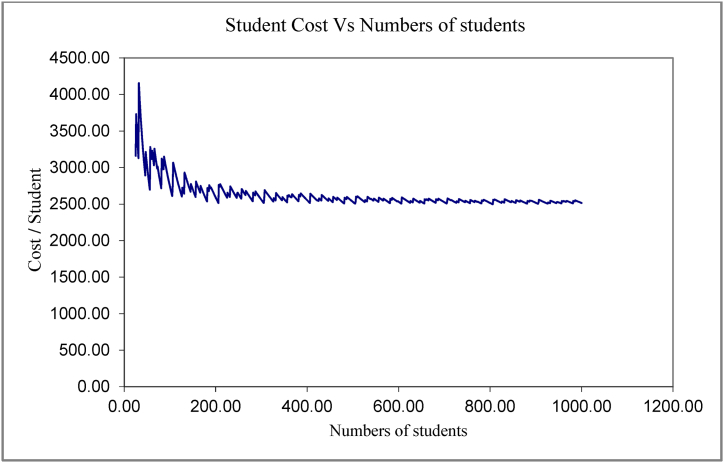
The relation between student cost and the numbers of the students.

In Fig. 5 the model show how the university's costing system behavior is simulated, where the figure represented the relation between the cost items and the number of the student, while Fig. 6 shows how the cost of the student decreases with the increase in the number of students, with an important note about the fluctuations, which may not be well illustrated in the graph, but indicate that there are convergent points in which the costs differ despite the contiguousness. how costs go through close fluctuations, and these fluctuations may not appear clearly as the graphic representation contains large values, so the difference between the values is not clear accurately, but if we look at Table No. (2), we notice the meaning of fluctuations.

**Fig. 7:**
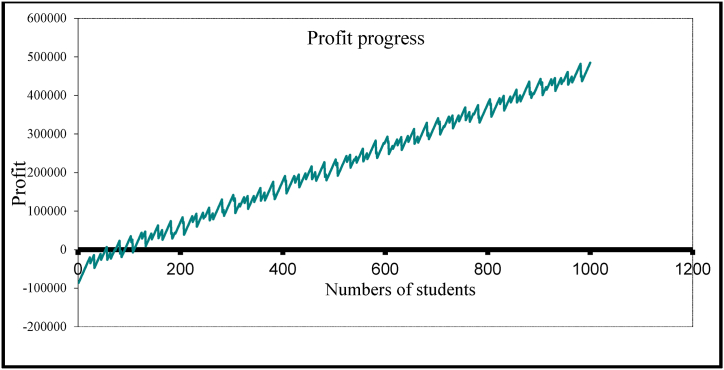
The profit and it relation with the student number.

The application also can provide other results such as finding the optimal profit or the suitable tuition fees. As example if we let the tuition fees for the student in the above example equal 3000$ per year (see Fig. 4 – the production price), then the application can provide results about the profit such as what is presented on Table 3 and Fig. 7.

**Table 3:** Selected values from the profits reports.

Numbers of students	Faculty	Assistants	Rooms	Lab	Total cost	Student's cost	The profit
33.00	2.00	2.00	1.00	1.00	143000.00	4333.33	−44000.00
53.00	2.00	3.00	1.00	1.00	161000.00	3037.74	−2000.00
54.00	2.00	3.00	1.00	1.00	161000.00	2981.48	1000.00
57.00	3.00	3.00	1.00	1.00	197000.00	3456.14	−26000.00
72.00	3.00	4.00	1.00	1.00	215000.00	2986.11	1000.00
80.00	3.00	4.00	2.00	1.00	220000.00	2750.00	20000.00
84.00	4.00	4.00	2.00	1.00	256000.00	3047.62	−4000.00
85.00	4.00	5.00	2.00	1.00	274000.00	3223.53	−19000.00
106.00	4.00	6.00	2.00	1.00	292000.00	2754.72	26000.00
107.00	5.00	6.00	2.00	1.00	328000.00	3065.42	−7000.00
124.00	5.00	6.00	2.00	1.00	328000.00	2645.16	44000.00
881.00	35.00	44.00	15.00	6.00	2207000.00	2505.11	436000.00
885.00	36.00	45.00	15.00	6.00	2261000.00	2554.80	394000.00
984.00	40.00	50.00	17.00	7.00	2515000.00	2555.89	437000.00
999.00	40.00	50.00	17.00	7.00	2515000.00	2517.52	482000.00
1000.00	40.00	50.00	17.00	7.00	2515000.00	2515.00	485000.00

## 6 Evaluation

The evaluation of the proposed approach can be assessed from three perspectives: model result accuracy and validation, model performance and efficiency, and a comparison study.

### 6.1 Result accuracy and validation

In terms of result accuracy and validation, the proposed model effectively simulates production cost items with their constraints within a linear programming framework. Given that our model represents the behaviors of production costs using linear equations, this implies that the model's results are both accurate and validated because linear programming models are known for their high accuracy and effectiveness across a wide range of optimization problems [25].

### 6.2 Model performance and efficiency

Regarding model performance and efficiency, our model formalizes the cost system's behavior using linear equations. This is significant because linear programming algorithms are known for their simplicity and high efficiency when coded, particularly in handling linear relationships and large datasets. Their scalability, robustness, and ability to provide optimal solutions make them superior to many other algorithms [26,27]. Additionally, our model is implemented as a computer system with database tables (as detailed in Section 5), achieving acceptable computational performance and efficiency. It can handle maximum data sizes while providing predictive results over extended periods.

### 6.3 Comparison with state-of-the-art

When comparing our work with the state-of-the-art, it is important to note that, although our experiment was conducted on an educational cost system, the same approach and designed computer system can be applied to various cost systems. Our work offers a comprehensive, standard solution with a flexible computer system platform that can simulate and optimize production costs for almost any production cost system, a feature not commonly found in previous studies as illustrated in Table 1. Most previous optimization work focuses on finding the marginal cost (the point at which costs equal returns). We did not find studies seeking to comprehensively determine the optimal use of product elements or the optimal cost conditions.

Studies attempting cost optimization either focused on special cases without comprehensive treatment [[18], [19], [20]] or provided a framework for quantitative analysis of specific problems [21,22]. Most studies offering computer simulation and optimization solutions either apply simulation algorithms to manage uncertainty or data analysis [13,17] or focus on specific activities without developing a comprehensive standard solution or addressing cost and production optimization in an ideal manner [10,11,14,28]. Additionally, many previous studies face complexity in model formulation or real-world representation [16,21,23] or lack scalability or generalization [10,11,15,17].

## 7 Conclusions

This paper makes a significant contribution to the field of simulating production costing behavior, optimizing costs, maximizing resource use, and providing economic return values and statistics. It presents an approach that expresses these relationships within a mathematical framework and implements them in a computer application. The experiment, applied to the university's college costing system as a case study, demonstrates that the computer application can be adapted to other costing systems. Designed as a platform for production costing systems, the results of our experiments align with the desired goals, confirming the model's success in finding ideal solutions.

## Declaration of competing interest

The author declare that they have no known competing financial interests or personal relationships that could have appeared to influence the work reported in this paper.
